# Cardiac sympatho-vagal balance and ventricular arrhythmia^☆^

**DOI:** 10.1016/j.autneu.2016.08.016

**Published:** 2016-08

**Authors:** Manish Kalla, Neil Herring, David J. Paterson

**Keywords:** Ventricular, Arrhythmia, Sympathetic, Vagus, Neuromodulation, Therapies

## Abstract

A hallmark of cardiovascular disease is cardiac autonomic dysregulation. The phenotype of impaired parasympathetic responsiveness and sympathetic hyperactivity in experimental animal models is also well documented in large scale human studies in the setting of heart failure and myocardial infarction, and is predictive of morbidity and mortality. Despite advances in emergency revascularisation strategies for myocardial infarction, device therapy for heart failure and secondary prevention pharmacotherapies, mortality from malignant ventricular arrhythmia remains high. Patients at highest risk or those with haemodynamically significant ventricular arrhythmia can be treated with catheter ablation and implantable cardioverter defibrillators, but the morbidity and reduction in quality of life due to the burden of ventricular arrhythmia and shock therapy persists. Therefore, future therapies must aim to target the underlying pathophysiology that contributes to the generation of ventricular arrhythmia. This review explores recent advances in mechanistic research in both limbs of the autonomic nervous system and potential avenues for translation into clinical therapy. In addition, we also discuss the relationship of these findings in the context of the reported efficacy of current neuromodulatory strategies in the management of ventricular arrhythmia.

## Highlights

• We review advances in mechanistic research in the cardiac autonomic nervous system.
• This is discussed in relation to neuromodulatory therapy for ventricular arrhythmia.
• Neuromodulation therapies can influence both neurotransmitters and co-transmitters.
• This may therefore improve on conventional medical treatment.

## 1 Introduction

A hallmark of cardiovascular disease (hypertension, myocardial infarction, heart failure and malignant ventricular arrhythmia) is cardiac autonomic dysregulation (Floras, 2003). The phenotype of impaired parasympathetic responsiveness and sympathetic hyperactivity in experimental animal models (Ma et al., 1997, Ishise et al., 1998, Sun et al., 1999, Motte et al., 2005) is also well documented in large scale human studies in the setting of heart failure and myocardial infarction, and is predictive of morbidity and mortality (La Rovere et al., 1998, Nolan et al., 1998). Despite advances in emergency revascularisation strategies for myocardial infarction, device therapy for heart failure and secondary prevention pharmacotherapies, mortality from malignant ventricular arrhythmia remains high. Patients at highest risk or those with haemodynamically significant ventricular arrhythmia can be treated with implantable cardioverter defibrillators (ICD) (AVID, 1997, Moss et al., 2002, Bardy et al., 2005), but the morbidity and reduction in quality of life due to the burden of ventricular arrhythmia and shock therapy persists. Therefore, future therapies must aim to target the underlying pathophysiology that contributes to the generation of ventricular arrhythmia. Emerging evidence now suggests that modulation of the autonomic nervous system with neuro-axis targeting is gaining utility as a novel therapy in this patient group (Ardell et al., 2016, Shivkumar et al., 2016).

## 2 The integrated heart-brain neuro-axis

The autonomic nervous system functions to tightly regulate cardiac excitability and contractile function (Habecker et al., 2016). The interconnected architecture has been elegantly explored and described by Ardell and Armour (Armour, 2008) based upon physiological data from multiple studies across varying spatial domains. This system is considered as a manifestation of three levels of neural hierarchy, moving away from the historical reciprocal thesis of cardiac control where the two arms of the ANS acted as ‘accelerator and brake’ on cardiac function. Instead, the excitability of cardiac parasympathetic pathways or sympathetic pathways depends on tonic inputs to synaptic junctions at several stages in the brain, spinal cord and in the extrinsic and intrinsic cardiac ganglia (Gebber et al., 1996, Kember et al., 2011, Fukuda et al., 2015). Level 1 encompasses the spinal cord and medulla with higher centre modulation (McAllen et al., 2011, Harper et al., 2013). Level 2 incorporates extracardiac neurons such as the stellate ganglia (Armour, 1986a, Armour, 1986b, Ardell et al., 2009) and level 3 includes all the intrinsic cardiac ganglia and nerves (Armour, 2008). Cardiac afferents and extracardiac circulatory receptors serve to transmit beat to beat sensory information to levels 1 and 2 and processing at these levels allows feedback loops which maintain physiological electrical and contractile stability in normal and stressed states (Ardell et al., 2016).

In the setting of cardiovascular disease or cardiac injury such as myocardial infarction, neurophysiological changes take place at distinct levels in the neural circuitry (Rubart and Zipes, 2005, Vaseghi and Shivkumar, 2008). Effects at the level of the organ such as scar formation and fibrosis contribute to heterogeneities in electrical activation and may contribute to the creation of fixed and functional substrate for re-entrant arrhythmia (Stevenson, 2009). There is also afferent mediated activation of neurohumoural systems and increased sympathetic stimulation and reduced vagal tone (Wang et al., 2014). In the short term, this is an adaptive response to maintain cardiac output (Kember et al., 2013), although at the cost of increased myocardial oxygen demand. However, following the acute injury phase, there is continued abnormal cardiac afferent signalling resulting in a maladaptive environment of persistent sympathetic activity (Zucker et al., 2012) that contributes to remodelling and the progression of cardiac disease that can ultimately lead to fatal arrhythmia.

Understanding of this complex cardiac neural-axis has led to targeted autonomic modulation therapies for heart failure and arrhythmia aimed at the cervical cardiac vagus and sympathetic nervous system respectively (Ardell et al., 2016). This review explores recent advances in mechanistic research in both limbs of the ANS and potential avenues for translation into clinical therapy. In addition, we also discuss the relationship of these findings in the context of the reported efficacy of current neuromodulatory strategies in the management of ventricular arrhythmia.

## 3 Mechanisms of ventricular arrhythmia

An understanding of the mechanisms responsible for the initiation and maintenance of ventricular arrhythmia is critical if effective treatment strategies are to be investigated and developed. The principle contributing factor to the onset of arrhythmia is re-entry. Fibrillation occurs when an electrical wave-break induces re-entry and leads to a sequence of new wave-breaks (Garfinkel et al., 2000, Weiss et al., 2000, Weiss et al., 2011). Wave-break is affected by static and dynamic factors and these influence the likelihood of local wave-break resulting in re-entry. Static factors are predominantly anatomical such as scar and fibrosis and lead to tissue heterogeneity and electrical remodelling that is fixed. Key components of dynamic factors are changes in membrane voltage and intracellular calcium, which are influenced by the autonomic nervous system and act synergistically with static factors to destabilise electrical activation (Weiss et al., 2000).

## 4 The effects of sympathetic stimulation on cardiac electrophysiology

Contemporary research has led to a spectrum of neuro-axial therapies in the setting of patients at high risk of malignant ventricular arrhythmia. This has been based on mechanistic evidence from models of sympathetic hyperactivity at the level of the organ in animals (Habecker et al., 2016), and more recently from human studies (Shivkumar et al., 2016).

The effect of sympathetic stimulation on global ventricular electrophysiology has been studied extensively in animal models (Mantravadi et al., 2007, Ng et al., 2009) and humans (Vaseghi et al., 2014). In the rabbit heart in-vitro, sympathetic stimulation using an electrode inserted in the spinal canal produces regional apex-base changes in restitution kinetics (Mantravadi et al., 2007, Ng et al., 2009). This is presumably mediated by regional differences sympathetic innervation and I_Ks_ distribution, potentially influencing the vulnerability to arrhythmia. The ability of local changes in sympathetic activity to trigger ventricular arrhythmias is well established (Nash et al., 2001). Epicardial injection of NE in the pig elicits triggered automaticity, and computational modelling implicated a Ca^2 +^ overload mechanism, supporting the hypothesis that heterogeneity or gradients of activation are pro-arrhythmic. The functional effect of post myocardial infarction remodelling has been elegantly demonstrated in patients undergoing endocardial and epicardial mapping as part of therapeutic catheter ablation procedures (Vaseghi et al., 2012). The effect of direct adrenergic stimulation with isoproterenol or reflex sympathetic stimulation in response to baroreflex activation elicites regional changes in repolarisation dynamics. This includes abnormal neural control in remote areas where a lack of action potential duration (APD) shortening in response to baroreflex activation suggests functional denervation. These results were reproduced in a porcine model of myocardial infarction following direct stellate ganglia stimulation (Ajijola et al., 2013). Interestingly, histological and molecular analysis of stellate ganglia tissue from these animals also demonstrates remodelling with increased tyrosine hydroxylase staining (Rajendran et al., 2016). This has been replicated in studies on stellate ganglia tissue from patients undergoing sympathectomy for refractory ventricular arrhythmia (Ajijola et al., 2012b).

## 5 Efferent cardiac sympathetic neurotransmission in health and disease

A growing body of work has demonstrated that sympathetic hyperactivity associated with several cardiovascular diseases resides, at least in part, with dysregulation in post-ganglionic cardiac sympathetic neurons. This is of particular interest as this area is perhaps more easily accessible for therapeutic intervention compared to the brainstem. Emerging evidence suggests that impaired NO and intracellular calcium handling are key intermediaries in sympathetic dyautonomia, since these abnormalities can be seen prior to the onset of the disease phenotype itself. For example, hypertension is associated with neurohumoral activation (Ely et al., 1997, Yemane et al., 2010) resulting in decreased parasympathetic responsiveness (Langewitz et al., 1994), sympathetic hyperactivity (Burns et al., 2007, Malpas, 2010, Grassi et al., 2015) and increased norepinephrine (NE) release (Rumantir et al., 2000). Post-ganglionic sympathetic neurons from the stellate ganglia of spontaneously hypertensive rats (SHRs) have a greater depolarisation-evoked intracellular Ca^2 +^ transient compared to normotensive controls. The Ca^2 +^ profile was observed in cells from neonatal, young pre-hypertensive and adult hypertensive animals suggesting the molecular phenotype is well conserved (Li et al., 2012). Moreover, the neuronal Ca^2 +^ current is increased in neurons from the SHR indicating that dysregulation of ion channel physiology contributes to the alteration in Ca^2 +^ transients (Lu et al., 2015). In addition, neuronal NE uptake by the NE uptake transporter (NET) is also decreased in stellate neurons from the SHR (Shanks et al., 2013a), thereby contributing to the increased NE spill over and heightened sympathetic responsiveness in both the adult (Herring et al., 2011) and young SHR before they develop high blood pressure (Shanks et al., 2013b). Of interest, the sympathetic phenotype can be rescued with site-specific nNOS gene transfer in tyrosine hydroxylase positive neurons (Wang et al., 2006) resulting in a reduction in Ca^2 +^ transients and inhibition of neurotransmission.

### 5.1 How does nNOS confer its protective effects?

Cytosolic nNOS translocates to caveolae post myocardial infarction and this is facilitated by its own shuttle adaptor protein, NOS1-AP/CAPON (Beigi et al., 2009). NOS1AP provides an interesting potential mechanistic link between cellular physiology and arrhythmia as GWAS studies have linked single nucleotide polymorphisms (SNPs) in the gene for NOS1-AP with QT variation (Arking et al., 2006) and sudden cardiac death (Westaway et al., 2011) in the general population. These SNPs are also risk factors for sudden cardiac death in patients with inherited long QT syndrome (Crotti et al., 2009, Tomas et al., 2010). NOS1AP is highly expressed in sympathetic neurons from the stellate neurons and is down regulated in the SHR (Lu et al., 2015). Overexpression of NOS1-AP in the SHR increases nNOS activity without a change in nNOS expression itself resulting in a reduction in neuronal Ca^2 +^ transients and NE release (Lu et al., 2015). These effects are abolished by nNOS inhibition, indicating a functional role for NOS1-AP in the NO mediated modulation of neurotransmission and therefore may represent a novel therapeutic target for modulating the gain of sympathetic activity in high risk populations (Li and Paterson, 2016).

### 5.2 Interaction with neurotrophic factors

It is interesting that sympathetic neuronal hyperactivity begins prior to the development of the disease phenotype and continues as the disease progresses. Sympathetic nerves project into the myocardium along with the parasympathetic innervation and together with sensory fibres act to tightly regulate cardiac function (Fukuda et al., 2015). The density of innervation corresponds to physiological triggers such as neurotrophins, of which nerve growth factor (NGF) is a prototypic member. NGF is upregulated in cardiac hypertrophy (Kimura et al., 2007) and also after myocardial infarction in a canine model (Zhou et al., 2004), leading to heterogeneous hyperinnervation. The relevance of this to arrhythmia was demonstrated by NGF infusion after myocardial infarction which resulted in an increased incidence of ventricular arrhythmia and sudden cardiac death (Cao et al., 2000). However, NGF also plays a role in the development of sensory innervation, and afferents responsible for the transduction of stimuli such as hypoxia, acidosis and pain during myocardial ischaemia (Hua et al., 2004). Sensory innervation has been shown to be impaired in conditions such as diabetes mellitus and may contribute to the phenomenon of silent myocardial ischaemia and the genesis of malignant ventricular arrhythmia (Faerman et al., 1977). Direct gene transfer of NGF into diabetic rat hearts improved cardiac sensory innervation (Ieda et al., 2006) and recombinant NGF has been shown to be safe when administered to diabetic patients with polyneuropathy (Apfel et al., 2000). Pathways such as this require further study to determine the contrasting effects on sensory innervation and potential deleterious effects of heterogeneous hyper-innervation seen post myocardial infarction. An alternative approach is to target the chondroitin sulfate proteoglycans present in scar tissue following MI which prevents sympathetic reinnervation by binding the neuronal protein tyrosine phosphatase receptor σ (PTPσ). Targeting PTPσ either genetically or pharmacologically restores sympathetic innervation to the scar and markedly reduces arrhythmia susceptibility in the immediate post-MI period (Gardner et al., 2015).

There are also chronic temporal changes in innervation that take place during disease progression in heart failure. Chronic sympathetic hyperactivity and exposure to NE leads to a reduction in NGF, and in severe decompensated heart failure, loss of innervation is seen (anatomic denervation). Heart failure is also associated with changes in other inflammatory and neurohumoural pathways including a range of growth factors and cytokines. Candidate molecules include leukemia inhibitory factor (LIF) and members of the interleukin (IL-6) family (Habecker et al., 2016). These molecules can induce changes in stellate ganglia neurons with increased expression of cholinergic markers via a gp130 signalling pathway (Kanazawa et al., 2010). This trans-differentiation process is of unknown significance, but there are data from murine studies of hypoxia induced heart failure in which sympathetic nerve specific gp130 knockout had adverse outcomes compared to controls indicating a potential protective role. A recent study also demonstrates neural remodelling including increased nNOS expression in a porcine model of chronic myocardial infarction model particularly in the ventral interventricular ganglionated plexi, dorsal root ganglia and stellate ganglia (Nakamura et al., 2016). The spatio-temporal features of changes in cardiac innervation continue to be the subject of study and may yield an alternative therapeutic approach in modifying the risk of ventricular arrhythmia.

## 6 The *anti*-arrhythmic effect of the vagus: historical perspective and emerging mechanistic insights

Efferent vagal innervation of the heart is classically thought to be only concentrated in the sinoatrial, atrial and atrioventricular nodal regions. Advances in anatomical techniques have however challenged the historical perspective that this innervation is restricted to the atria and structures in the conduction system (Randall, 1984, Levy and Martin, 1996). Immunohistochemical techniques identifying either acetylcholinesterase (AChE) and more recently choline acetyltransferase (ChAT) have led to revisions in this dogma, where significant cholinergic epicardial and endocardial innervation of atria and ventricles across a range of mammalian species has been observed (Blomquist et al., 1987, Pauza et al., 2002, Batulevicius et al., 2005, Ulphani et al., 2010). There is however considerable species variation in sites of preganglionic nerve terminations in parasympathetic ganglia (Pardini et al., 1987, Singh et al., 1996, Coote, 2013) and this may underlie some of the species dependent variation in response to VNS reported in the literature. Despite a sparse innervation of the ventricle in a variety of species, functionally VNS can produce a decrease in left ventricular contractility in human subjects (Lewis et al., 2001).

The *anti*-fibrillatory effect of the vagus was first described in 1859 (Einbrodt, 1859) and elegant studies have demonstrated that VNS decreases the occurrence of ventricular arrhythmia during CAO, ischaemia-reperfusion and also in the absence of structural heart disease (Kent et al., 1973, Corr and Gillis, 1974, Myers et al., 1974, Kolman et al., 1975, Yoon et al., 1977). The most compelling demonstration of the anti-fibrillatory effect of VNS and the importance of vagal tone has come from Peter Schwartz and colleagues. They employed a canine model of healed anterior myocardial infarction at increased risk of malignant ventricular arrhythmia. Animals were subjected to increasing workload on a treadmill post-infarction with concurrent occlusion of the circumflex artery resulting in VF in 60% of animals (Schwartz et al., 1984, Schwartz et al., 1988). Animals that survived this stressor had increased vagal tone indicated by a reduction in heart rate during exercise, and this protective effect was abolished by muscarinic blockade with atropine. Direct VNS in the same model system also resulted in a significant reduction in VF occurrence (Vanoli et al., 1991).

The mechanistic basis of this *anti*-fibrillatory property of VNS can be considered in terms of the effect on static and dynamic contributors to the development of ventricular arrhythmias. Tissue heterogeneity due to scar and fibrosis after myocardial infarction is a key substrate for the initiation and maintenance of re-entry and ventricular tachycardia (Stevenson, 2009). Uniform activation of the ventricle is dependent upon gap junction distribution and function. Alteration in gap junction expression is seen in the context of ischaemia, HF and cardiomyopathy and these groups are at high risk of ventricular arrhythmia. Ando et al. (Ando et al., 2005) demonstrated that VNS resulted in maintenance of gap junction function as evidenced by preservation of phosphorylated connexin 43 and an 87% reduction in post myocardial infarction ventricular tachycardia (VT) in rats. Similar results have been reported by Sabbah (Sabbah, 2011) where chronic VNS resulted in normalisation of connexin 43 expression in a canine model of micro-embolisation induced HF. These pre-clinical observations of positive remodelling of substrate by VNS are exciting observations and clinical studies with VNS in HF patients may be able to assess this effect via complex imaging studies. Inflammatory pathways play a key role in fibrosis (Nicoletti and Michel, 1999), scar formation and hypertrophy (Klein et al., 2000). VNS in the canine HF model normalises levels of IL-6 and TNFα (Wang et al., 2003) and chronic VNS reduces plasma levels of angiotensin II (Zhang et al., 2009), another potent pro-fibrotic mediator.

### 6.1 The direct and indirect effect of VNS on sympathetic signalling and a direct effect on ventricular electrophysiology

Stimulating the cardiac vagus results in bradycardia and some studies have suggested that the *anti*-arrhythmic effect is attenuated if the heart rate is controlled (Goldstein et al., 1973, Kent et al., 1973, Kent et al., 1974, Myers et al., 1974, Zuanetti et al., 1987), although recent data (Brack et al., 2011, Kalla et al., 2016) has demonstrated that an anti-fibrillatory property of cholinergic signalling persists independent of heart rate. Nitric oxide (NO) plays a key role in mediating vagal bradycardia and its mechanism is site-specific (Paton et al., 2002). Depolarisation of nerve terminals leads to release of acetycholine (ACh) which binds to sino-atrial node (SAN) cell muscarinic receptors (M_2_) coupled to inhibitory G proteins (Schulz et al., 2005). This leads to hyperpolarisation via stimulation of ACh-activated K^+^ channels, reduction in adenylate cyclase (and therefore cAMP), with subsequent decrease in the hyperpolarisation-activated current, I_f_, and PKA dependent phosphorylation of the L-type calcium current, I_CaL_, thereby reducing diastolic depolarisation and heart rate (Lakatta et al., 2010). Neuronal NOS (nNOS) is thought to be the critical source of NO in modulating vagal neurotransmission. This mechanism was suggested by the observation that NO donors (Herring and Paterson, 2001) and inhibitors of NOS and soluble guanylyl cyclase (sGC) (Herring et al., 2000) do not affect the heart rate response to ACh in vitro. In addition, application of ACh to atria from nNOS knockout mice also remains intact despite impaired vagal responses (Choate et al., 2001). Release of radio-labelled ACh from field stimulated atria is also increased by NO donors and abolished by sGC inhibitors (Herring and Paterson, 2001), further supporting a pre-synaptic, autocrine role rather than direct co-transmitter function in terms of vagal control of heart rate. Mechanistically, NO augments parasympathetic transmission by augmenting pre-synaptic cAMP-PKA regulation of N- and P-type calcium channels by a cGMP-PDE3 mediated increase in cAMP which phosphorylates N-type channels leading to an increase in ACh release (Herring et al., 2002).

Recent data has demonstrated that the *anti*-fibrillatory effect of direct VNS is independent of heart rate but still dependent on NO (Brack et al., 2011). The potential for a direct effect of VNS on ventricular electrophysiology has been studied in a series of experiments utilising a Langendorff perfused rabbit heart with intact bilateral vagal innervation. Left or right VNS flattened the action potential duration (APD) restitution curve and prolonged ventricular effective refractory period (ERP) (Ng et al., 2001) in keeping with the anti-arrhythmic mechanism suggested by the restitution hypothesis (Weiss et al., 2005). A more defined anti-fibrillatory effect was also demonstrated by determining ventricular fibrillation threshold (VFT) to remove the confounding effect of heart rate reduction. VFT was increased with left or right VNS despite differential heart rate reductions seen from each limb (Ng et al., 2007), and in contrast to the earlier studies of VNS, these observations were seen in the absence of background sympathetic stimulation. This would support a direct ventricular effect rather than dependence on indirect ‘accentuated antagonism’ of adrenergic signalling. The pattern of ventricular repolarisation was also reversed with bilateral VNS (Ng et al., 2007), although the mechanistic significance of this remains uncertain and requires further investigation.

The dogma of vagal or cholinergic signalling has been that release of ACh from cholinergic nerve terminals results in activation of M_2_ ACh receptors (AChR) and downstream coupling to G_i_, eNOS and G_k_ signalling cascades. G_i_ inhibits adenylyl cyclase and thereby reduces cAMP dependent stimulation of L-type Ca^2 +^ current (LTCC) and phosholamban. M_2_ dependent stimulation of eNOS activity has also been proposed as a mechanism for accentuated antagonism of β-receptor stimulation when cAMP levels are already elevated (Balligand et al., 1993, Han et al., 1995). Generation of NO via this pathway may increase sGC generation of cGMP via stimulation of PDE2 (Han et al., 1998). The role of the pathway is controversial (Herring et al., 2002) and many groups have observed antagonism of heart rate and contractility to be intact despite genetic deletion of eNOS (Martin et al., 2006). M_2_ receptor activation of G_k_ increases I_KACh_ and many propose that this is the main mechanism by which cholinergic accentuated antagonism is mediated (Mesirca et al., 2013).

In the innervated, rabbit heart preparation described above, the *anti*-fibrillatory effect of direct left or right VNS was interestingly maintained in the presence of atropine, but abolished with non-specific NOS inhibition and restored with supplementation of l-arginine, the substrate for NOS (Brack et al., 2007, Brack et al., 2011). This suggests a non-receptor dependent, nitrergic anti-arrhythmic mechanism of VNS. The release of NO was confirmed with an increase in epicardial DAF-2 fluorescence. The effect of VNS on APD restitution was also lost with NOS inhibition, but the effect on ERP was maintained, suggesting divergent pathways mediating these effects on ventricular electrophysiology. Central modulation of rat vagal signalling via optogenetic silencing of preganglioninc neurons from the dorsal ventral motor nucleus (Mastitskaya et al., 2012) demonstrates some beneficial effects on electrophysiology. During central silencing of DVMN with combined β-blockade and muscarinic blockade, reductions in ventricular ERP and VT could be abolished by NOS blockade with 7-nitroinidazole (Machhada et al., 2015) suggestive of paracrine NO signalling. Others however, observe contrasting results (Kalla et al., 2016). Using an isolated rat heart preparation, stimulation with an ACh analogue, carbamylcholine (CCh) that is resistant to cholinesterase, results in an increase in VFT independent of heart rate. CCh perfusion also resulted in prolongation of APD during fixed rate pacing and flattening of the APD restitution curve that is consistent with an *anti*-arrhythmic effect. This rise in VFT was also dependent upon the generation of NO as the effect was abolished by non-selective and neuronal NOS blockade and downstream blockade of sGC. CCh perfusion also increased NO metabolites (NOx), and this was prevented by mecamylamine, a specific inhibitor of the nicotinic AChR demonstrating that the source of NO was neuronal.

Therefore, observations in the rat are in keeping with the observations in the rabbit that nNOS derived NO is a key component of the anti-arrhythmic effect of cholinergic signalling. However, in the rat the anti-fibrillatory effect is abolished by atropine in keeping with the established autocrine role of NO (Herring and Paterson, 2001). Furthermore, an NO donor, can increase VFT, an effect abolished by atropine, indicating that NO was acting upstream of the muscarinic receptor. The most likely mechanistic explanation of these observations is the nNOS derived NO from parasympathetic ganglia facilitates ACh release from sites of ganglionic projections via a cGMP-PDE3 dependent pathway increasing PKA dependent phosphorylation of N-type calcium channels (Herring and Paterson, 2001), but this may be dependent on the density of innervation and the expression or activity of the nNOS enzyme.

Notwithstanding the above observations, these data must also be considered within the limitations of the model systems studied. Sensory fibres make up to 70% of the cervical vagus (Berthoud and Neuhuber, 2000) and they will also be recruited with direct stimulation of the transected nerve and may influence the generation of nNOS derived NO. The relevance of afferent fibre stimulation has been assessed in a porcine model of VNS with and without nerve transection (Yamakawa et al., 2015). Here VNS without decentralisation activates afferent fibres in the ipsilateral vagus nerve with accompanying reflex inhibition of cardiac parasympathetic efferent electrophysiological and haemodynamic effects. Furthermore, the species dependent variation in parasympathetic projections may also contribute to the relative importance of the two proposed pathways. However, the critical role of muscarinic receptors is supported by the CCh experiments and this is in keeping with established literature (Corr and Gillis, 1974, Yoon et al., 1977, Vanoli et al., 1991, De Ferrari et al., 1992, De Ferrari et al., 1993) and recent studies of VNS in ischaemia-reperfusion (Shinlapawittayatorn et al., 2014).

The *anti*-arrhythmic role of NO in mediating vagal protection is supported by studies across multiple domains, and future studies will need to further delineate the sites of action and optimum strategies to exploit this effect therapeutically. Speculatively, a nicotinic receptor based NO mediated mechanism that converges on the muscarinic receptor is conceivably amenable to a more targeted approach with gene therapy or a pharmacological treatment strategy. Percutaneous cardiac gene transfer of nNOS increases vagal neurotransmission and bradycardia (Mohan et al., 2002) and reduces mortality three days post myocardial infarction in the guinea pig (Dawson et al., 2008). Whether gene transfer of nNOS into cardiac cholinergic ganglia provides a direct *anti*-fibrillatory action on the ventricle that could be used therapeutically remains to be established, although gene transfer of nNOS into the pig cervical vagus increases cardiac baroreflex sensitivity (Heaton et al., 2005).

## 7 Neuromodulation targeting the cervical vagus nerve

Targeting the cardiac vagus with device therapy for heart failure is a recent example of a translational bench to bedside research. The first in man trial of vagus nerve stimulation (VNS) (De Ferrari et al., 2011) was a small, proof of concept study that demonstrated an improvement in symptoms in patients with advanced heart failure treated with VNS. However, larger, clinical studies such as ANTHEM-HF (Premchand et al., 2014) and NECTAR-HR (Zannad et al., 2015) have yielded disappointing results in terms of echocardiographic and clinical end points. A larger, randomised clinical trial, INOVATE-HF (Hauptman et al., 2012) has also recently reported no impact on heart failure events or mortality in patients with ejection fractions < 40% and New York Heart Association (NYHA) class III symptoms (Gold et al., 2016). It is worth highlighting that these trials employed a variety of different stimulation currents and frequencies, different stimulation timings (continuous v′ R wave synchronised bursts) as well as different approaches (left verses right) all of which can impact on the efficacy of the treatment (Kember et al., 2014, Ardell et al., 2015). Trials to date have focused on cardiac imaging and heart failure symptoms, but of considerable mechanistic interest may be the potential role for appropriate VNS in suppressing ventricular arrhythmia.

## 8 Neuromodulation directly targeting the cardiac sympathetic nervous system

The potential of the vagus nerve as a therapeutic target in heart failure continues to be the subject of clinical trials, but challenges remain in delivering effective device therapy. Historically, pharmacological approaches such as cholinesterase inhibition (Behling et al., 2003, Santos-Almeida et al., 2015) have also been limited. Therefore the sympatho-adrenal axis has been the principal target for established pharmacotherapies such as β-blockers and ACE inhibitors, with both achieving significant reductions in morbidity and mortality (ISIS-1, 1986, CONSENSUS, 1987, Pfeffer et al., 1992, CIBIS-II, 1999). However, sudden cardiac death (SCD) remains a significant clinical problem and sympathetic activation is a key contributor in patients with and without structural heart disease. Recent observations have led to neuro-axial therapies directly targeting the sympathetic nervous system in the setting of VT due to structural heart disease and channelopathies (Shen and Zipes, 2014). Patients can present with VT that is recurrent or incessant, resulting in haemodynamic compromise, multiple ICD shocks and high mortality (Verma et al., 2004). Conventional management strategies include trials of anti-arrhythmic medications and catheter ablation (Pedersen et al., 2014), but recurrence of arrhythmia is common (Tung et al., 2015). Neuromodulation by directly targeting the sympathetic nervous system has demonstrated efficacy in animal and human studies and the commonest strategies employed are thoracic epidural anaesthesia and cardiac sympathetic denervation by stellectomy (CSD) (Mahajan et al., 2005, Ajijola et al., 2012a). In the largest case series to date, 41 patients underwent left or bilateral stellectomy for refractory ventricular arrhythmia and failed catheter ablation (Vaseghi et al., 2014). The majority of patients were taking anti-arrhythmic drugs and 73% were taking β-blockers. CSD resulted in almost 50% of patients being free of ICD shocks at 1 year with the remaining experiencing a significant reduction in arrhythmic events. A similar approach has been used successfully in long QT syndrome (Moss and McDonald, 1971, Schwartz et al., 1991) and catecholaminergic polymorphic VT (Collura et al., 2009). In a series of 17 patients with LQTS or CPVT undergoing left CSD for recurrent arrhythmia despite β-blockade in all, 87% of patients had a significant reduction in arrhythmic events following LCSD. In the setting of VT storm, thoracic epidural anaesthesia (Mahajan et al., 2005) which acts to modify sympathetic outflow rather than remove neurotransmitter source, has also demonstrated efficacy.

## 9 Indirect neuromodulation of cardiac sympatho-vagal balance

Other approaches to reducing cardiac sympathetic drive include targeting higher centres via deep brain stimulation (DBS). DBS of the periaqueductal grey is a treatment for chronic pain can also lead to increases or decreases in blood pressure depending on the location of the electrode (Green et al., 2005, Pereira et al., 2010)and alter heart rate variability (Pereira et al., 2010) and baroreflex sensitivity by influencing sympathetic outflow (Sverrisdottir et al., 2014). Spinal cord stimulation at the level of T1–3 not only reduces cardiac sympathetic drive but also targets neural processing via intrathoracic extra-cardiac and intrinsic cardiac ganglia, as well as local circuit neurons (Ardell et al., 2016). In early human trials, continuous spinal cord stimulation improved symptoms and left ventricular dimensions in patients with severe symptomatic heart failure (Tse et al., 2015) but not when administered intermittently (Zipes et al., 2016). Afferent signalling can also be targeted via denervating the carotid body (McBryde et al., 2013, Schultz et al., 2015) or via renal sympathetic nerve ablation (Esler, 2015) in order to reduce cardiac sympathetic drive. Renal denervation can have beneficial effects on cardiac electrophysiology in animal models (Huang et al., 2014) and has been used to treat VT storm in patients (Remo et al., 2014), despite the disappointing results of the Symplicity-HTN3 trial in drug resistant hypertension (Bhatt et al., 2014).

## 10 Conclusions

Although neuromodulation therapies offer promise in terms of treatment of ventricular arrhythmias, it is interesting to note that most to date have been trialed in patients already taking the maximally tolerated doses of β-blockers (Herring, 2014). The mechanistic basis of neuromodulation therapies may be found in emerging evidence of the role of factors produced within the microenvironment of the heart, its vasculature and between neuronal populations influencing local sympatho-vagal balance. These neuromodulators may reside within neurones (such as nNOS and NOS1-AP) or be mediators that are co-transmitted with NE such as ATP, neuropeptide Y (NPY) and galanin, or ACh such as vasoactive intestinal peptide (VIP). NPY and galanin have been shown to be released with high-level sympathetic nerve stimulation and reduce cholinergic ACh release and bradycardia (Herring et al., 2008, Herring et al., 2012). In addition NPY may act directly on ventricular myocytes to trigger arrhythmias independent of beta-receptor stimulation (Herring, 2015). Cardiac myocytes and the vasculature also release a variety of mediators such as angiotensin II and natriuretic peptides which may influence neuronal physiology in a paracrine manner (Herring and Paterson, 2009), and these crosstalk and paracrine pathways converge on neuronal and myocyte Ca^2 +^ handling, thereby potentially influencing arrhythmogenesis. Neuromodulation therapies may not only remove the source of the principal neurotransmitter, but also influence the local release of co-transmitters providing increased efficacy over conventional medical therapies. The functional significance of these pathways and potential therapeutics avenues in disease states is yet to be established.

## Conflicts of interest

None.

## References

[bb0005] Ajijola O.A., Vaseghi M., Mahajan A., Shivkumar K. (2012). Bilateral cardiac sympathetic denervation: why, who and when?. Expert. Rev. Cardiovasc. Ther..

[bb0010] Ajijola O.A., Wisco J.J., Lambert H.W., Mahajan A., Stark E., Fishbein M.C., Shivkumar K. (2012). Extracardiac neural remodeling in humans with cardiomyopathy. Circ. Arrhythm. Electrophysiol..

[bb0015] Ajijola O.A., Yagishita D., Patel K.J., Vaseghi M., Zhou W., Yamakawa K., So E., Lux R.L., Mahajan A., Shivkumar K. (2013). Focal myocardial infarction induces global remodeling of cardiac sympathetic innervation: neural remodeling in a spatial context. Am. J. Physiol. Heart Circ. Physiol..

[bb0020] Ando M., Katare R.G., Kakinuma Y., Zhang D., Yamasaki F., Muramoto K., Sato T. (2005). Efferent vagal nerve stimulation protects heart against ischemia-induced arrhythmias by preserving connexin43 protein. Circulation.

[bb0025] Apfel S.C., Schwartz S., Adornato B.T., Freeman R., Biton V., Rendell M., Vinik A., Giuliani M., Stevens J.C., Barbano R., Dyck P.J. (2000). Efficacy and safety of recombinant human nerve growth factor in patients with diabetic polyneuropathy: a randomized controlled trial. rhNGF Clinical Investigator Group. JAMA.

[bb0030] Ardell J.L., Cardinal R., Vermeulen M., Armour J.A. (2009). Dorsal spinal cord stimulation obtunds the capacity of intrathoracic extracardiac neurons to transduce myocardial ischemia. Am. J. Physiol. Regul. Integr. Comp. Physiol..

[bb0035] Ardell J.L., Rajendran P.S., Nier H.A., KenKnight B.H., Armour J.A. (2015). Central-peripheral neural network interactions evoked by vagus nerve stimulation: functional consequences on control of cardiac function. Am. J. Physiol. Heart Circ. Physiol..

[bb0040] Ardell J.L., Andresen M.C., Armour J.A., Billman G.E., Chen P.S., Foreman R.D., Herring N., O'Leary D.S., Sabbah H.N., Schultz H.D., Sunagawa K., Zucker I.H. (2016). Translational neurocardiology: preclinical models and cardioneural integrative aspects. J. Physiol..

[bb0045] Arking D.E., Pfeufer A., Post W., Kao W.H., Newton-Cheh C., Ikeda M., West K., Kashuk C., Akyol M., Perz S., Jalilzadeh S., Illig T., Gieger C., Guo C.Y., Larson M.G., Wichmann H.E., Marban E., O'Donnell C.J., Hirschhorn J.N., Kaab S., Spooner P.M., Meitinger T., Chakravarti A. (2006). A common genetic variant in the NOS1 regulator NOS1AP modulates cardiac repolarization. Nat. Genet..

[bb0050] Armour J.A. (1986). Activity of in situ stellate ganglion neurons of dogs recorded extracellularly. Can. J. Physiol. Pharmacol..

[bb0055] Armour J.A. (1986). Neuronal activity recorded extracellularly in chronically decentralized in situ canine middle cervical ganglia. Can. J. Physiol. Pharmacol..

[bb0060] Armour J.A. (2008). Potential clinical relevance of the ‘little brain’ on the mammalian heart. Exp. Physiol..

[bb0065] AVID (1997). A comparison of antiarrhythmic-drug therapy with implantable defibrillators in patients resuscitated from near-fatal ventricular arrhythmias. The Antiarrhythmics Versus Implantable Defibrillators (AVID) Investigators. N. Engl. J. Med..

[bb0070] Balligand J.L., Kelly R.A., Marsden P.A., Smith T.W., Michel T. (1993). Control of cardiac muscle cell function by an endogenous nitric oxide signaling system. Proc. Natl. Acad. Sci..

[bb0075] Bardy G.H., Lee K.L., Mark D.B., Poole J.E., Packer D.L., Boineau R., Domanski M., Troutman C., Anderson J., Johnson G., McNulty S.E., Clapp-Channing N., Davidson-Ray L.D., Fraulo E.S., Fishbein D.P., Luceri R.M., Ip J.H. (2005). Amiodarone or an implantable cardioverter-defibrillator for congestive heart failure. N. Engl. J. Med..

[bb0080] Batulevicius D., Pauziene N., Pauza D.H. (2005). Architecture and age-related analysis of the neuronal number of the guinea pig intrinsic cardiac nerve plexus. Ann. Anat..

[bb0085] Behling A., Moraes R.S., Rohde L.E., Ferlin E.L., Nobrega A.C., Ribeiro J.P. (2003). Cholinergic stimulation with pyridostigmine reduces ventricular arrhythmia and enhances heart rate variability in heart failure. Am. Heart J..

[bb0090] Beigi F., Oskouei B.N., Zheng M., Cooke C.A., Lamirault G., Hare J.M. (2009). Cardiac nitric oxide synthase-1 localization within the cardiomyocyte is accompanied by the adaptor protein, CAPON. Nitric Oxide.

[bb0095] Berthoud H.R., Neuhuber W.L. (2000). Functional and chemical anatomy of the afferent vagal system. Auton. Neurosci..

[bb0100] Bhatt D.L., Kandzari D.E., O'Neill W.W., D'Agostino R., Flack J.M., Katzen B.T., Leon M.B., Liu M., Mauri L., Negoita M., Cohen S.A., Oparil S., Rocha-Singh K., Townsend R.R., Bakris G.L. (2014). A controlled trial of renal denervation for resistant hypertension. N. Engl. J. Med..

[bb0105] Blomquist T.M., Priola D.V., Romero A.M. (1987). Source of intrinsic innervation of canine ventricles: a functional study. Am. J. Phys..

[bb0110] Brack K.E., Patel V.H., Coote J.H., Ng G.A. (2007). Nitric oxide mediates the vagal protective effect on ventricular fibrillation via effects on action potential duration restitution in the rabbit heart. J. Physiol..

[bb0115] Brack K.E., Coote J.H., Ng G.A. (2011). Vagus nerve stimulation protects against ventricular fibrillation independent of muscarinic receptor activation. Cardiovasc. Res..

[bb0120] Burns J., Sivananthan M.U., Ball S.G., Mackintosh A.F., Mary D.A., Greenwood J.P. (2007). Relationship between central sympathetic drive and magnetic resonance imaging-determined left ventricular mass in essential hypertension. Circulation.

[bb0125] Cao J.M., Chen L.S., KenKnight B.H., Ohara T., Lee M.H., Tsai J., Lai W.W., Karagueuzian H.S., Wolf P.L., Fishbein M.C., Chen P.S. (2000). Nerve sprouting and sudden cardiac death. Circ. Res..

[bb0130] Choate J.K., Danson E.J., Morris J.F., Paterson D.J. (2001). Peripheral vagal control of heart rate is impaired in neuronal NOS knockout mice. Am. J. Physiol. Heart Circ. Physiol..

[bb0135] CIBIS-II (1999). The cardiac insufficiency Bisoprolol study II (CIBIS-II): A randomised trial. Lancet.

[bb0140] Collura C.A., Johnson J.N., Moir C., Ackerman M.J. (2009). Left cardiac sympathetic denervation for the treatment of long QT syndrome and catecholaminergic polymorphic ventricular tachycardia using video-assisted thoracic surgery. Heart Rhythm..

[bb0145] CONSENSUS (1987). Effects of enalapril on mortality in severe congestive heart failure. Results of the Cooperative North Scandinavian Enalapril Survival Study (CONSENSUS). The CONSENSUS Trial Study Group. N. Engl. J. Med..

[bb0150] Coote J.H. (2013). Myths and realities of the cardiac vagus. J. Physiol..

[bb0155] Corr P.B., Gillis R.A. (1974). Role of the vagus nerves in the cardiovascular changes induced by coronary occlusion. Circulation.

[bb0160] Crotti L., Monti M.C., Insolia R., Peljto A., Goosen A., Brink P.A., Greenberg D.A., Schwartz P.J., George A.L. (2009). NOS1AP is a genetic modifier of the long-QT syndrome. Circulation.

[bb0165] Dawson T.A., Li D., Woodward T., Barber Z., Wang L., Paterson D.J. (2008). Cardiac cholinergic NO-cGMP signaling following acute myocardial infarction and nNOS gene transfer. Am. J. Physiol. Heart Circ. Physiol..

[bb0170] De Ferrari G.M., Vanoli E., Curcuruto P., Tommasini G., Schwartz P.J. (1992). Prevention of life-threatening arrhythmias by pharmacologic stimulation of the muscarinic receptors with oxotremorine. Am. Heart J..

[bb0175] De Ferrari G.M., Salvati P., Grossoni M., Ukmar G., Vaga L., Patrono C., Schwartz P.J. (1993). Pharmacologic modulation of the autonomic nervous system in the prevention of sudden cardiac death. A study with propranolol, methacholine and oxotremorine in conscious dogs with a healed myocardial infarction. J. Am. Coll. Cardiol..

[bb0180] De Ferrari G.M., Crijns H.J., Borggrefe M., Milasinovic G., Smid J., Zabel M., Gavazzi A., Sanzo A., Dennert R., Kuschyk J., Raspopovic S., Klein H., Swedberg K., Schwartz P.J. (2011). Chronic vagus nerve stimulation: a new and promising therapeutic approach for chronic heart failure. Eur. Heart J..

[bb0185] Einbrodt (1859). UeberHerzreizungundihrVerhaeltniszum Blutdruck. Akademie der Wissenschaften (Vienna).

[bb0190] Ely D., Caplea A., Dunphy G., Daneshvar H., Turner M., Milsted A., Takiyyudin M. (1997). Spontaneously hypertensive rat Y chromosome increases indexes of sympathetic nervous system activity. Hypertension.

[bb0195] Esler M. (2015). Renal denervation for treatment of drug-resistant hypertension. Trends Cardiovasc. Med..

[bb0200] Faerman I., Faccio E., Milei J., Nunez R., Jadzinsky M., Fox D., Rapaport M. (1977). Autonomic neuropathy and painless myocardial infarction in diabetic patients. Histologic evidence of their relationship. Diabetes.

[bb0205] Floras J.S. (2003). Sympathetic activation in human heart failure: diverse mechanisms, therapeutic opportunities. Acta Physiol. Scand..

[bb0210] Fukuda K., Kanazawa H., Aizawa Y., Ardell J.L., Shivkumar K. (2015). Cardiac innervation and sudden cardiac death. Circ. Res..

[bb0215] Gardner R.T., Wang L., Lang B.T., Cregg J.M., Dunbar C.L., Woodward W.R., Silver J., Ripplinger C.M., Habecker B.A. (2015). Targeting protein tyrosine phosphatase sigma after myocardial infarction restores cardiac sympathetic innervation and prevents arrhythmias. Nat. Commun..

[bb0220] Garfinkel A., Kim Y.H., Voroshilovsky O., Qu Z., Kil J.R., Lee M.H., Karagueuzian H.S., Weiss J.N., Chen P.S. (2000). Preventing ventricular fibrillation by flattening cardiac restitution. Proc. Natl. Acad. Sci..

[bb0225] Gebber G.L., Zhong S., Paitel Y. (1996). Bispectral analysis of complex patterns of sympathetic nerve discharge. Am. J. Physiol..

[bb0230] Gold M.R., Van Veldhuisen D.J., Hauptman P.J., Borggrefe M., Kubo S.H., Lieberman R.A., Milasinovic G., Berman B.J., Djordjevic S., Neelagaru S., Schwartz P.J., Starling R.C., Mann D.L. (2016). Vagus nerve stimulation for the treatment of heart failure: the INOVATE-HF trial. J. Am. Coll. Cardiol..

[bb0235] Goldstein R.E., Karsh R.B., Smith E.R., Orlando M., Norman D., Farnham G., Redwood D.R., Epstein S.E. (1973). Influence of atropine and of vagally mediated bradycardia on the occurrence of ventricular arrhythmias following acute coronary occlusion in closed-chest dogs. Circulation.

[bb0240] Grassi G., Mark A., Esler M. (2015). The sympathetic nervous system alterations in human hypertension. Circ. Res..

[bb0245] Green A.L., Wang S., Owen S.L., Xie K., Liu X., Paterson D.J., Stein J.F., Bain P.G., Aziz T.Z. (2005). Deep brain stimulation can regulate arterial blood pressure in awake humans. Neuroreport.

[bb0250] Habecker B.A., Anderson M.E., Birren S.J., Fukuda K., Herring N., Hoover D.B., Kanazawa H., Paterson D.J., Ripplinger C.M. (2016). Molecular and cellular neurocardiology: development, cellular and molecular adaptations to heart disease. J. Physiol..

[bb0255] Han X., Shimoni Y., Giles W.R. (1995). A cellular mechanism for nitric oxide-mediated cholinergic control of mammalian heart rate. J. Gen. Physiol..

[bb0260] Han X., Kubota I., Feron O., Opel D.J., Arstall M.A., Zhao Y.Y., Huang P., Fishman M.C., Michel T., Kelly R.A. (1998). Muscarinic cholinergic regulation of cardiac myocyte ICa-L is absent in mice with targeted disruption of endothelial nitric oxide synthase. Proc. Natl. Acad. Sci..

[bb0265] Harper R.M., Kumar R., Ogren J.A., Macey P.M. (2013). Sleep-disordered breathing: effects on brain structure and function. Respir. Physiol. Neurobiol..

[bb0270] Hauptman P.J., Schwartz P.J., Gold M.R., Borggrefe M., Van Veldhuisen D.J., Starling R.C., Mann D.L. (2012). Rationale and study design of the increase of vagal tone in heart failure study: INOVATE-HF. Am. Heart J..

[bb0275] Heaton D.A., Golding S., Bradley C.P., Dawson T.A., Cai S., Channon K.M., Paterson D.J. (2005). Targeted nNOS gene transfer into the cardiac vagus rapidly increases parasympathetic function in the pig. J. Mol. Cell. Cardiol..

[bb0280] Herring N. (2014). The kidney-heart connection during electrical storm: from bedside back to bench. Exp. Physiol..

[bb0285] Herring N. (2015). Autonomic control of the heart: going beyond the classical neurotransmitters. Exp. Physiol..

[bb0290] Herring N., Paterson D.J. (2001). Nitric oxide-cGMP pathway facilitates acetylcholine release and bradycardia during vagal nerve stimulation in the guinea-pig in vitro. J. Physiol..

[bb0295] Herring N., Paterson D.J. (2009). Neuromodulators of peripheral cardiac sympatho-vagal balance. Exp. Physiol..

[bb0300] Herring N., Golding S., Paterson D.J. (2000). Pre-synaptic NO-cGMP pathway modulates vagal control of heart rate in isolated adult guinea pig atria. J. Mol. Cell. Cardiol..

[bb0305] Herring N., Danson E.J., Paterson D.J. (2002). Cholinergic control of heart rate by nitric oxide is site specific. News Physiol. Sci..

[bb0310] Herring N., Lokale M.N., Danson E.J., Heaton D.A., Paterson D.J. (2008). Neuropeptide Y reduces acetylcholine release and vagal bradycardia via a Y2 receptor-mediated, protein kinase C-dependent pathway. J. Mol. Cell. Cardiol..

[bb0315] Herring N., Lee C.W., Sunderland N., Wright K., Paterson D.J. (2011). Pravastatin normalises peripheral cardiac sympathetic hyperactivity in the spontaneously hypertensive rat. J. Mol. Cell. Cardiol..

[bb0320] Herring N., Cranley J., Lokale M.N., Li D., Shanks J., Alston E.N., Girard B.M., Carter E., Parsons R.L., Habecker B.A., Paterson D.J. (2012). The cardiac sympathetic co-transmitter galanin reduces acetylcholine release and vagal bradycardia: implications for neural control of cardiac excitability. J. Mol. Cell. Cardiol..

[bb0325] Hua F., Harrison T., Qin C., Reifsteck A., Ricketts B., Carnel C., Williams C.A. (2004). C-Fos expression in rat brain stem and spinal cord in response to activation of cardiac ischemia-sensitive afferent neurons and electrostimulatory modulation. Am. J. Physiol. Heart Circ. Physiol..

[bb0330] Huang B., Yu L., He B., Lu Z., Wang S., He W., Yang K., Liao K., Zhang L., Jiang H. (2014). Renal sympathetic denervation modulates ventricular electrophysiology and has a protective effect on ischaemia-induced ventricular arrhythmia. Exp. Physiol..

[bb0335] Ieda M., Kanazawa H., Ieda Y., Kimura K., Matsumura K., Tomita Y., Yagi T., Onizuka T., Shimoji K., Ogawa S., Makino S., Sano M., Fukuda K. (2006). Nerve growth factor is critical for cardiac sensory innervation and rescues neuropathy in diabetic hearts. Circulation.

[bb0340] Ishise H., Asanoi H., Ishizaka S., Joho S., Kameyama T., Umeno K., Inoue H. (1998). Time course of sympathovagal imbalance and left ventricular dysfunction in conscious dogs with heart failure. J. Appl. Physiol..

[bb0345] ISIS-1 (1986). Randomised trial of intravenous atenolol among 16 027 cases of suspected acute myocardial infarction: ISIS-1. First International Study of Infarct Survival Collaborative Group. Lancet.

[bb0350] Kalla M., Chotalia M., Coughlan C., Hao G., Crabtree M.J., Tomek J., Bub G., Paterson D.J., Herring N. (2016). Protection against ventricular fibrillation via cholinergic receptor stimulation and the generation of nitric oxide. J. Physiol..

[bb0355] Kanazawa H., Ieda M., Kimura K., Arai T., Kawaguchi-Manabe H., Matsuhashi T., Endo J., Sano M., Kawakami T., Kimura T., Monkawa T., Hayashi M., Iwanami A., Okano H., Okada Y., Ishibashi-Ueda H., Ogawa S., Fukuda K. (2010). Heart failure causes cholinergic transdifferentiation of cardiac sympathetic nerves via gp130-signaling cytokines in rodents. J. Clin. Invest..

[bb0360] Kember G., Armour J.A., Zamir M. (2011). Neural control of heart rate: the role of neuronal networking. J. Theor. Biol..

[bb0365] Kember G., Armour J.A., Zamir M. (2013). Neural control hierarchy of the heart has not evolved to deal with myocardial ischemia. Physiol. Genomics.

[bb0370] Kember G., Ardell J.L., Armour J.A., Zamir M. (2014). Vagal nerve stimulation therapy: what is being stimulated?. PLoS One.

[bb0375] Kent K.M., Smith E.R., Redwood D.R., Epstein S.E. (1973). Electrical stability of acutely ischemic myocardium. Influences of heart rate and vagal stimulation. Circulation.

[bb0380] Kent K.M., Smith E.R., Redwood D.R., Epstein S.E. (1974). Beneficial electrophysiologic effects of nitroglycerin during acute myocardial infarction. Am. J. Cardiol..

[bb0385] Kimura K., Ieda M., Kanazawa H., Yagi T., Tsunoda M., Ninomiya S., Kurosawa H., Yoshimi K., Mochizuki H., Yamazaki K., Ogawa S., Fukuda K. (2007). Cardiac sympathetic rejuvenation: a link between nerve function and cardiac hypertrophy. Circ. Res..

[bb0390] Klein R.M., Vester E.G., Brehm M.U., Dees H., Picard F., Niederacher D., Beckmann M.W., Strauer B.E. (2000). Inflammation of the myocardium as an arrhythmia trigger. Z. Kardiol..

[bb0395] Kolman B.S., Verrier R.L., Lown B. (1975). The effect of vagus nerve stimulation upon vulnerability of the canine ventricle: role of sympathetic-parasympathetic interactions. Circulation.

[bb0400] La Rovere M.T., Bigger J.T., Marcus F.I., Mortara A., Schwartz P.J. (1998). Baroreflex sensitivity and heart-rate variability in prediction of total cardiac mortality after myocardial infarction. ATRAMI (Autonomic Tone and Reflexes After Myocardial Infarction) Investigators. Lancet.

[bb0405] Lakatta E.G., Maltsev V.A., Vinogradova T.M. (2010). A coupled SYSTEM of intracellular Ca2 + clocks and surface membrane voltage clocks controls the timekeeping mechanism of the heart's pacemaker. Circ. Res..

[bb0410] Langewitz W., Ruddel H., Schachinger H. (1994). Reduced parasympathetic cardiac control in patients with hypertension at rest and under mental stress. Am. Heart J..

[bb0415] Levy M.N., Martin P.J., Shepherd J.T., Vatner S.F. (1996). Autonomic control of cardiac conduction and automaticity. Nervous Control of the Heart.

[bb0420] Lewis M.E., Al-Khalidi A.H., Bonser R.S., Clutton-Brock T., Morton D., Paterson D., Townend J.N., Coote J.H. (2001). Vagus nerve stimulation decreases left ventricular contractility in vivo in the human and pig heart. J. Physiol..

[bb0425] Li D., Paterson D.J. (2016). Cyclic nucleotide regulation of cardiac sympatho-vagal responsiveness. J. Physiol..

[bb0430] Li D., Lee C.W., Buckler K., Parekh A., Herring N., Paterson D.J. (2012). Abnormal intracellular calcium homeostasis in sympathetic neurons from young prehypertensive rats. Hypertension.

[bb0435] Lu C.J., Hao G., Nikiforova N., Larsen H.E., Liu K., Crabtree M.J., Li D., Herring N., Paterson D.J. (2015). CAPON modulates neuronal calcium handling and cardiac sympathetic neurotransmission during dysautonomia in hypertension. Hypertension.

[bb0440] Ma R., Zucker I.H., Wang W. (1997). Central gain of the cardiac sympathetic afferent reflex in dogs with heart failure. Am. J. Phys..

[bb0445] Machhada A., Ang R., Ackland G.L., Ninkina N., Buchman V.L., Lythgoe M.F., Trapp S., Tinker A., Marina N., Gourine A.V. (2015). Control of ventricular excitability by neurons of the dorsal motor nucleus of the vagus nerve. Heart Rhythm..

[bb0450] Mahajan A., Moore J., Cesario D.A., Shivkumar K. (2005). Use of thoracic epidural anesthesia for management of electrical storm: a case report. Heart Rhythm..

[bb0455] Malpas S.C. (2010). Sympathetic nervous system overactivity and its role in the development of cardiovascular disease. Physiol. Rev..

[bb0460] Mantravadi R., Gabris B., Liu T., Choi B.R., de Groat W.C., Ng G.A., Salama G. (2007). Autonomic nerve stimulation reverses ventricular repolarization sequence in rabbit hearts. Circ. Res..

[bb0465] Martin S.R., Emanuel K., Sears C.E., Zhang Y.H., Casadei B. (2006). Are myocardial eNOS and nNOS involved in the beta-adrenergic and muscarinic regulation of inotropy? A systematic investigation. Cardiovasc. Res..

[bb0470] Mastitskaya S., Marina N., Gourine A., Gilbey M.P., Spyer K.M., Teschemacher A.G., Kasparov S., Trapp S., Ackland G.L., Gourine A.V. (2012). Cardioprotection evoked by remote ischaemic preconditioning is critically dependent on the activity of vagal pre-ganglionic neurones. Cardiovasc. Res..

[bb0475] McAllen R.M., Salo L.M., Paton J.F., Pickering A.E. (2011). Processing of central and reflex vagal drives by rat cardiac ganglion neurones: an intracellular analysis. J. Physiol..

[bb0480] McBryde F.D., Abdala A.P., Hendy E.B., Pijacka W., Marvar P., Moraes D.J., Sobotka P.A., Paton J.F. (2013). The carotid body as a putative therapeutic target for the treatment of neurogenic hypertension. Nat. Commun..

[bb0485] Mesirca P., Marger L., Toyoda F., Rizzetto R., Audoubert M., Dubel S., Torrente A.G., Difrancesco M.L., Muller J.C., Leoni A.L., Couette B., Nargeot J., Clapham D.E., Wickman K., Mangoni M.E. (2013). The G-protein-gated K + channel, IKACh, is required for regulation of pacemaker activity and recovery of resting heart rate after sympathetic stimulation. J. Gen. Physiol..

[bb0490] Mohan R.M., Heaton D.A., Danson E.J., Krishnan S.P., Cai S., Channon K.M., Paterson D.J. (2002). Neuronal nitric oxide synthase gene transfer promotes cardiac vagal gain of function. Circ. Res..

[bb0495] Moss A.J., McDonald J. (1971). Unilateral cervicothoracic sympathetic ganglionectomy for the treatment of long QT interval syndrome. N. Engl. J. Med..

[bb0500] Moss A.J., Zareba W., Hall W.J., Klein H., Wilber D.J., Cannom D.S., Daubert J.P., Higgins S.L., Brown M.W., Andrews M.L. (2002). Prophylactic implantation of a defibrillator in patients with myocardial infarction and reduced ejection fraction. N. Engl. J. Med..

[bb0505] Motte S., Mathieu M., Brimioulle S., Pensis A., Ray L., Ketelslegers J.M., Montano N., Naeije R., van de Borne P., Entee K.M. (2005). Respiratory-related heart rate variability in progressive experimental heart failure. Am. J. Physiol. Heart Circ. Physiol..

[bb0510] Myers R.W., Pearlman A.S., Hyman R.M., Goldstein R.A., Kent K.M., Goldstein R.E., Epstein S.E. (1974). Beneficial effects of vagal stimulation and bradycardia during experimental acute myocardial ischemia. Circulation.

[bb0515] Nakamura K., Ajijola O.A., Aliotta E., Armour J.A., Ardell J.L., Shivkumar K. (2016). Pathological effects of chronic myocardial infarction on peripheral neurons mediating cardiac neurotransmission. Auton. Neurosci..

[bb0520] Nash M.P., Thornton J.M., Sears C.E., Varghese A., O'Neill M., Paterson D.J. (2001). Ventricular activation during sympathetic imbalance and its computational reconstruction. J. Appl. Physiol..

[bb0525] Ng G.A., Brack K.E., Coote J.H. (2001). Effects of direct sympathetic and vagus nerve stimulation on the physiology of the whole heart–a novel model of isolated Langendorff perfused rabbit heart with intact dual autonomic innervation. Exp. Physiol..

[bb0530] Ng G.A., Brack K.E., Patel V.H., Coote J.H. (2007). Autonomic modulation of electrical restitution, alternans and ventricular fibrillation initiation in the isolated heart. Cardiovasc. Res..

[bb0535] Ng G.A., Mantravadi R., Walker W.H., Ortin W.G., Choi B.R., de Groat W., Salama G. (2009). Sympathetic nerve stimulation produces spatial heterogeneities of action potential restitution. Heart Rhythm..

[bb0540] Nicoletti A., Michel J.B. (1999). Cardiac fibrosis and inflammation: interaction with hemodynamic and hormonal factors. Cardiovasc. Res..

[bb0545] Nolan J., Batin P.D., Andrews R., Lindsay S.J., Brooksby P., Mullen M., Baig W., Flapan A.D., Cowley A., Prescott R.J., Neilson J.M., Fox K.A. (1998). Prospective study of heart rate variability and mortality in chronic heart failure: results of the United Kingdom heart failure evaluation and assessment of risk trial (UK-heart). Circulation.

[bb0550] Pardini B.J., Patel K.P., Schmid P.G., Lund D.D. (1987). Location, distribution and projections of intracardiac ganglion cells in the rat. J. Auton. Nerv. Syst..

[bb0555] Paton J.F., Kasparov S., Paterson D.J. (2002). Nitric oxide and autonomic control of heart rate: a question of specificity. Trends Neurosci..

[bb0560] Pauza D.H., Pauziene N., Pakeltyte G., Stropus R. (2002). Comparative quantitative study of the intrinsic cardiac ganglia and neurons in the rat, guinea pig, dog and human as revealed by histochemical staining for acetylcholinesterase. Ann. Anat..

[bb0565] Pedersen C.T., Kay G.N., Kalman J., Borggrefe M., Della-Bella P., Dickfeld T., Dorian P., Huikuri H., Kim Y.H., Knight B., Marchlinski F., Ross D., Sacher F., Sapp J., Shivkumar K., Soejima K., Tada H., Alexander M.E., Triedman J.K., Yamada T., Kirchhof P., Lip G.Y., Kuck K.H., Mont L., Haines D., Indik J., Dimarco J., Exner D., Iesaka Y., Savelieva I. (2014). EHRA/HRS/APHRS expert consensus on ventricular arrhythmias. Heart Rhythm..

[bb0570] Pereira E.A., Lu G., Wang S., Schweder P.M., Hyam J.A., Stein J.F., Paterson D.J., Aziz T.Z., Green A.L. (2010). Ventral periaqueductal grey stimulation alters heart rate variability in humans with chronic pain. Exp. Neurol..

[bb0575] Pfeffer M.A., Braunwald E., Moye L.A., Basta L., Brown E.J., Cuddy T.E., Davis B.R., Geltman E.M., Goldman S., Flaker G.C. (1992). Effect of captopril on mortality and morbidity in patients with left ventricular dysfunction after myocardial infarction. Results of the survival and ventricular enlargement trial. The SAVE Investigators. N. Engl. J. Med..

[bb0580] Premchand R.K., Sharma K., Mittal S., Monteiro R., Dixit S., Libbus I., DiCarlo L.A., Ardell J.L., Rector T.S., Amurthur B., KenKnight B.H., Anand I.S. (2014). Autonomic regulation therapy via left or right cervical vagus nerve stimulation in patients with chronic heart failure: results of the ANTHEM-HF trial. J. Card. Fail..

[bb0585] Rajendran P.S., Nakamura K., Ajijola O.A., Vaseghi M., Armour J.A., Ardell J.L., Shivkumar K. (2016). Myocardial infarction induces structural and functional remodelling of the intrinsic cardiac nervous system. J. Physiol..

[bb0590] Randall W. (1984). Nervous Control of Cardiovascular Function.

[bb0595] Remo B.F., Preminger M., Bradfield J., Mittal S., Boyle N., Gupta A., Shivkumar K., Steinberg J.S., Dickfeld T. (2014). Safety and efficacy of renal denervation as a novel treatment of ventricular tachycardia storm in patients with cardiomyopathy. Heart Rhythm..

[bb0600] Rubart M., Zipes D.P. (2005). Mechanisms of sudden cardiac death. J. Clin. Invest..

[bb0605] Rumantir M.S., Kaye D.M., Jennings G.L., Vaz M., Hastings J.A., Esler M.D. (2000). Phenotypic evidence of faulty neuronal norepinephrine reuptake in essential hypertension. Hypertension.

[bb0610] Sabbah H.N. (2011). Electrical vagus nerve stimulation for the treatment of chronic heart failure. Cleve. Clin. J. Med..

[bb0615] Santos-Almeida F.M., Girao H., da Silva C.A., Salgado H.C., Fazan R. (2015). Cholinergic stimulation with pyridostigmine protects myocardial infarcted rats against ischemic-induced arrhythmias and preserves connexin43 protein. Am. J. Physiol. Heart Circ. Physiol..

[bb0620] Schultz H.D., Marcus N.J., Del Rio R. (2015). Role of the carotid body Chemoreflex in the pathophysiology of heart failure: a perspective from animal studies. Adv. Exp. Med. Biol..

[bb0625] Schulz R., Rassaf T., Massion P.B., Kelm M., Balligand J.L. (2005). Recent advances in the understanding of the role of nitric oxide in cardiovascular homeostasis. Pharmacol. Ther..

[bb0630] Schwartz P.J., Billman G.E., Stone H.L. (1984). Autonomic mechanisms in ventricular fibrillation induced by myocardial ischemia during exercise in dogs with healed myocardial infarction. An experimental preparation for sudden cardiac death. Circulation.

[bb0635] Schwartz P.J., Vanoli E., Stramba-Badiale M., De Ferrari G.M., Billman G.E., Foreman R.D. (1988). Autonomic mechanisms and sudden death. New insights from analysis of baroreceptor reflexes in conscious dogs with and without a myocardial infarction. Circulation.

[bb0640] Schwartz P.J., Locati E.H., Moss A.J., Crampton R.S., Trazzi R., Ruberti U. (1991). Left cardiac sympathetic denervation in the therapy of congenital long QT syndrome. A worldwide report. Circulation.

[bb0645] Shanks J., Mane S., Ryan R., Paterson D.J. (2013). Ganglion-specific impairment of the norepinephrine transporter in the hypertensive rat. Hypertension.

[bb0650] Shanks J., Manou-Stathopoulou S., Lu C.J., Li D., Paterson D.J., Herring N. (2013). Cardiac sympathetic dysfunction in the prehypertensive spontaneously hypertensive rat. Am. J. Physiol. Heart Circ. Physiol..

[bb0655] Shen M.J., Zipes D.P. (2014). Role of the autonomic nervous system in modulating cardiac arrhythmias. Circ. Res..

[bb0660] Shinlapawittayatorn K., Chinda K., Palee S., Surinkaew S., Kumfu S., Kumphune S., Chattipakorn S., KenKnight B.H., Chattipakorn N. (2014). Vagus nerve stimulation initiated late during ischemia, but not reperfusion, exerts cardioprotection via amelioration of cardiac mitochondrial dysfunction. Heart Rhythm..

[bb0665] Shivkumar K., Ajijola O.A., Anand I., Armour J.A., Chen P.S., Esler M., De Ferrari G., Fishbein M.C., Goldberger J.J., Harper R.M., Joyner M.J., Khalsa S.S., Kumar R., Lane R., Mahajan A., Po S., Schwartz P.J., Somers V.K., Valderrabano M., Vaseghi M., Zipes D.P. (2016). Clinical neurocardiology-defining the value of neuroscience-based cardiovascular therapeutics. J. Physiol..

[bb0670] Singh S., Johnson P.I., Lee R.E., Orfei E., Lonchyna V.A., Sullivan H.J., Montoya A., Tran H., Wehrmacher W.H., Wurster R.D. (1996). Topography of cardiac ganglia in the adult human heart. J. Thorac. Cardiovasc. Surg..

[bb0675] Stevenson W.G. (2009). Ventricular scars and ventricular tachycardia. Trans. Am. Clin. Climatol. Assoc..

[bb0680] Sun S.Y., Wang W., Zucker I.H., Schultz H.D. (1999). Enhanced activity of carotid body chemoreceptors in rabbits with heart failure: role of nitric oxide. J. Appl. Physiol..

[bb0685] Sverrisdottir Y.B., Green A.L., Aziz T.Z., Bahuri N.F., Hyam J., Basnayake S.D., Paterson D.J. (2014). Differentiated baroreflex modulation of sympathetic nerve activity during deep brain stimulation in humans. Hypertension.

[bb0690] Tomas M., Napolitano C., De Giuli L., Bloise R., Subirana I., Malovini A., Bellazzi R., Arking D.E., Marban E., Chakravarti A., Spooner P.M., Priori S.G. (2010). Polymorphisms in the NOS1AP gene modulate QT interval duration and risk of arrhythmias in the long QT syndrome. J. Am. Coll. Cardiol..

[bb0695] Tse H.F., Turner S., Sanders P., Okuyama Y., Fujiu K., Cheung C.W., Russo M., Green M.D., Yiu K.H., Chen P., Shuto C., Lau E.O., Siu C.W. (2015). Thoracic Spinal Cord Stimulation for Heart Failure as a Restorative Treatment (SCS HEART study): first-in-man experience. Heart Rhythm..

[bb0700] Tung R., Vaseghi M., Frankel D.S., Vergara P., Di Biase L., Nagashima K., Yu R., Vangala S., Tseng C.H., Choi E.K., Khurshid S., Patel M., Mathuria N., Nakahara S., Tzou W.S., Sauer W.H., Vakil K., Tedrow U., Burkhardt J.D., Tholakanahalli V.N., Saliaris A., Dickfeld T., Weiss J.P., Bunch T.J., Reddy M., Kanmanthareddy A., Callans D.J., Lakkireddy D., Natale A., Marchlinski F., Stevenson W.G., Della Bella P., Shivkumar K. (2015). Freedom from recurrent ventricular tachycardia after catheter ablation is associated with improved survival in patients with structural heart disease: an International VT Ablation Center Collaborative Group study. Heart Rhythm..

[bb0705] Ulphani J.S., Cain J.H., Inderyas F., Gordon D., Gikas P.V., Shade G., Mayor D., Arora R., Kadish A.H., Goldberger J.J. (2010). Quantitative analysis of parasympathetic innervation of the porcine heart. Heart Rhythm..

[bb0710] Vanoli E., De Ferrari G.M., Stramba-Badiale M., Hull S.S., Foreman R.D., Schwartz P.J. (1991). Vagal stimulation and prevention of sudden death in conscious dogs with a healed myocardial infarction. Circ. Res..

[bb0715] Vaseghi M., Shivkumar K. (2008). The role of the autonomic nervous system in sudden cardiac death. Prog. Cardiovasc. Dis..

[bb0720] Vaseghi M., Lux R.L., Mahajan A., Shivkumar K. (2012). Sympathetic stimulation increases dispersion of repolarization in humans with myocardial infarction. Am. J. Physiol. Heart Circ. Physiol..

[bb0725] Vaseghi M., Gima J., Kanaan C., Ajijola O.A., Marmureanu A., Mahajan A., Shivkumar K. (2014). Cardiac sympathetic denervation in patients with refractory ventricular arrhythmias or electrical storm: intermediate and long-term follow-up. Heart Rhythm..

[bb0730] Verma A., Kilicaslan F., Marrouche N.F., Minor S., Khan M., Wazni O., Burkhardt J.D., Belden W.A., Cummings J.E., Abdul-Karim A., Saliba W., Schweikert R.A., Tchou P.J., Martin D.O., Natale A. (2004). Prevalence, predictors, and mortality significance of the causative arrhythmia in patients with electrical storm. J. Cardiovasc. Electrophysiol..

[bb0735] Wang H., Yu M., Ochani M., Amella C.A., Tanovic M., Susarla S., Li J.H., Yang H., Ulloa L., Al-Abed Y., Czura C.J., Tracey K.J. (2003). Nicotinic acetylcholine receptor alpha7 subunit is an essential regulator of inflammation. Nature.

[bb0740] Wang L., Li D., Plested C.P., Dawson T., Teschemacher A.G., Paterson D.J. (2006). Noradrenergic neuron-specific overexpression of nNOS in cardiac sympathetic nerves decreases neurotransmission. J. Mol. Cell. Cardiol..

[bb0745] Wang H.J., Wang W., Cornish K.G., Rozanski G.J., Zucker I.H. (2014). Cardiac sympathetic afferent denervation attenuates cardiac remodeling and improves cardiovascular dysfunction in rats with heart failure. Hypertension.

[bb0750] Weiss J.N., Chen P.S., Qu Z., Karagueuzian H.S., Garfinkel A. (2000). Ventricular fibrillation: how do we stop the waves from breaking?. Circ. Res..

[bb0755] Weiss J.N., Qu Z., Chen P.S., Lin S.F., Karagueuzian H.S., Hayashi H., Garfinkel A., Karma A. (2005). The dynamics of cardiac fibrillation. Circulation.

[bb0760] Weiss J.N., Nivala M., Garfinkel A., Qu Z. (2011). Alternans and arrhythmias: from cell to heart. Circ. Res..

[bb0765] Westaway S.K., Reinier K., Huertas-Vazquez A., Evanado A., Teodorescu C., Navarro J., Sinner M.F., Gunson K., Jui J., Spooner P., Kaab S., Chugh S.S. (2011). Common variants in CASQ2, GPD1L, and NOS1AP are significantly associated with risk of sudden death in patients with coronary artery disease. Circ. Cardiovasc. Genet..

[bb0770] Yamakawa K., Rajendran P.S., Takamiya T., Yagishita D., So E.L., Mahajan A., Shivkumar K., Vaseghi M. (2015). Vagal nerve stimulation activates vagal afferent fibers that reduce cardiac efferent parasympathetic effects. Am. J. Physiol. Heart Circ. Physiol..

[bb0775] Yemane H., Busauskas M., Burris S.K., Knuepfer M.M. (2010). Neurohumoral mechanisms in deoxycorticosterone acetate (DOCA)-salt hypertension in rats. Exp. Physiol..

[bb0780] Yoon M.S., Han J., Tse W.W., Rogers R. (1977). Effects of vagal stimulation, atropine, and propranolol on fibrillation threshold of normal and ischemic ventricles. Am. Heart J..

[bb0785] Zannad F., De Ferrari G.M., Tuinenburg A.E., Wright D., Brugada J., Butter C., Klein H., Stolen C., Meyer S., Stein K.M., Ramuzat A., Schubert B., Daum D., Neuzil P., Botman C., Castel M.A., D'Onofrio A., Solomon S.D., Wold N., Ruble S.B. (2015). Chronic vagal stimulation for the treatment of low ejection fraction heart failure: results of the NEural Cardiac TherApy foR Heart Failure (NECTAR-HF) randomized controlled trial. Eur. Heart J..

[bb0790] Zhang Y., Popovic Z.B., Bibevski S., Fakhry I., Sica D.A., Van Wagoner D.R., Mazgalev T.N. (2009). Chronic vagus nerve stimulation improves autonomic control and attenuates systemic inflammation and heart failure progression in a canine high-rate pacing model. Circ. Heart Fail..

[bb0795] Zhou S., Chen L.S., Miyauchi Y., Miyauchi M., Kar S., Kangavari S., Fishbein M.C., Sharifi B., Chen P.S. (2004). Mechanisms of cardiac nerve sprouting after myocardial infarction in dogs. Circ. Res..

[bb0800] Zipes D.P., Neuzil P., Theres H., Caraway D., Mann D.L., Mannheimer C., Van Buren P., Linde C., Linderoth B., Kueffer F., Sarazin S.A., DeJongste M.J., Investigators D.-H.T. (2016). Determining the feasibility of spinal cord neuromodulation for the treatment of chronic systolic heart failure: the DEFEAT-HF study. JACC Heart Fail..

[bb0805] Zuanetti G., De Ferrari G.M., Priori S.G., Schwartz P.J. (1987). Protective effect of vagal stimulation on reperfusion arrhythmias in cats. Circ. Res..

[bb0810] Zucker I.H., Patel K.P., Schultz H.D. (2012). Neurohumoral stimulation. Heart Fail. Clin..

